# Gut microbiota in cats with inflammatory bowel disease and low-grade intestinal T-cell lymphoma

**DOI:** 10.3389/fmicb.2024.1346639

**Published:** 2024-05-15

**Authors:** Amandine Drut, Héla Mkaouar, Aicha Kriaa, Vincent Mariaule, Nizar Akermi, Tristan Méric, Odile Sénécat, Emmanuelle Maguin, Juan Hernandez, Moez Rhimi

**Affiliations:** ^1^MIHA Team, INRAE, AgroParisTech, Micalis Institute, Université Paris-Saclay, Jouy-en-Josas, France; ^2^Oniris VetAgroBio Nantes, Université de Nantes, Nantes, France

**Keywords:** cat, inflammatory bowel diseases, low-grade intestinal T-cell lymphoma, gut microbiota, chronic enteropathies

## Abstract

In cats and humans, several physiological and environmental factors have been shown to alter the gut microbiota of healthy individuals. Cats share several diseases with humans such as inflammatory bowel diseases and low-grade intestinal T-cell lymphoma. The physiopathology of these chronic enteropathies is poorly understood but may involve disequilibrium of the gut microbiota composition and disruption of normal microbiome activity profiles. These disorders are increasingly diagnosed in the feline species due to improved medicalization and easier access to endoscopy in veterinary practice. This review addresses the current data on the gut microbiota of cats in health and in chronic enteropathies. Such functional analysis will help the advancement of innovative diagnostic tools and targeted therapeutic strategies.

## 1 Introduction

Domestic cats, residing in 29% of North American households in 2022, hold a distinguished place as beloved pets (Scott Nolen, 2022). Their owners maintain elevated expectations for their healthcare. In feline medicine, chronic enteropathies (CE), comprising Inflammatory Bowel Diseases (IBD) and Low-Grade Intestinal T-Cell Lymphoma (LGITL), are major concerns with increasingly high incidence (Marsilio, 2021). Both incurable diseases display challenging diagnosis and available treatments have significant adverse effects (Marsilio, 2021). Moreover, LGITL is thought to evolve toward higher-grade lymphoma despite appropriate treatment in some cats (Wright et al., 2019). Therefore, there is still a need to understand the pathophysiology of feline CE to set out new therapeutic strategies. Many spontaneous feline diseases have their counterpart in human medicine, which highlights their interest in comparative pathology. Recently, LGITL has been described as an interesting model of indolent T-cell lymphoproliferative disorder of the gastrointestinal tract (ITLPD-GI) in humans (Freiche et al., 2021). Advances in this biomedical field are likely to benefit to feline patients but also to provide new insights into human diseases.

Like humans, the digestive tract of cats hosts a myriad of microorganisms whose links with physiology and health have been established. In humans, the gut microbiota interacts with the immune system and has been identified as a major player in the pathogenesis of IBD, comprising Crohn’s disease (CD) and ulcerative colitis (UC), and various gastrointestinal neoplasms including gastric mucosa-associated lymphoid tissue lymphoma (Nakamura et al., 1998; Nishida et al., 2018). Pet cats share the domestic environment of their owners and are therefore exposed to the same environmental factors. Additionally, they are in daily contact with their owners and share resting and cooking areas, which may promote the fecal-oral transmission of commensals and enteropathogenic bacteria in both directions, as suspected with *Clostridioides difficile* (Alves et al., 2022). Cat ownership has been shown to induce bacterial fluxes in humans and influence owners’ gut microbiota composition and function (Du et al., 2021).

The domestic cat (*Felis silvestris catus*) is a strict carnivore but can also digest, absorb and metabolize dietary carbohydrates. Despite this major difference with the human dietary regimen, feline models remain relevant for the study of the gut microbiota and intestinal health in humans, regarding the wide variation in meat consumption in human populations and the possible association between excessive intake of red meat or overprocessed meat and colorectal neoplasms (Farvid et al., 2021). The special dietary requirements of cats impact the profile of their core gut microbiome (Ganz et al., 2022). Several recent studies have focused on the composition of the feline gut microbiota in health and CE (Garraway et al., 2018; Marsilio et al., 2019; Ganz et al., 2022). Therefore, we aim in this review to discuss the links between gut microbiota, intestinal inflammation and the development of IBD and LGITL in cats, in order to highlight the gaps in knowledge and propose future research perspectives.

## 2 Gut microbiota in healthy cats

The cat’s gut microbiota is teeming with a variety of microorganisms including bacteria, fungi, viruses, and protozoa. Bacteria overwhelmingly dominate the cats’ microbiota, comprising roughly 98% of the total population and playing crucial roles in maintaining host health (Pilla and Suchodolski, 2021). The role of the fungal population, or mycobiota, and its contribution to health and disease in the feline gut remains elusive (Tay et al., 2022), as well as the implication of protozoa and viruses in the microbiota community.

When examining the bacterial composition, several studies of the feline gut microbiota have identified five phyla: *Actinobacteria*, *Bacteroidetes*, *Firmicutes*, *Fusobacteria*, and *Proteobacteria* (Ritchie et al., 2008, 2010; Tun et al., 2012; Ganz et al., 2022). Two additional studies recovered only four of these bacterial phyla and failed to detect *Fusobacteria* and *Proteobacteria*, respectively (Desai et al., 2009; Handl et al., 2011). The five bacterial phyla identified in cats are shared with the core microbiome of humans and pet dogs (Arumugam et al., 2011; You and Kim, 2021). In cats, two studies have identified the Bacteroidetes/Chlorobi group as prominent (Tun et al., 2012; Ganz et al., 2022), with *Bacteroides* species such as *B. vulgatus*, *B. thetaiotaomicron*, *B. fragilis* and *Porphyromonas gingivalis* being the most common and widespread (Tun et al., 2012). Firmicutes also prevail (Ritchie et al., 2008, 2010; Desai et al., 2009; Handl et al., 2011), with Clostridia being the most abundant class, along with Erysipelotrichia, Negativicutes (Ganz et al., 2022), Bacilli, Mollicutes, and Lactobacillales (Tun et al., 2012). Lactobacillales comprise the most important probiotic bacteria of the cat, dog and human gut microbiome, and has great beneficial effects on their intestinal health (Strompfová et al., 2017; Fusi et al., 2019; Rastogi and Singh, 2022). In the widest study aimed at establishing a comprehensive dataset for the “core microbiota” in 161 North American domestic cats, the taxonomic analysis identified 30 major genera belonging to the five predominant phyla listed above (Ganz et al., 2022). Detected genera were noted in over 55% of the healthy cat population and comprised *Prevotella*, *Bacteroides*, *Collinsella*, *Catenibacterium*, *Blautia*, *Faecalibacterium* and *Megasphaera* which displayed the highest relative abundance. Of these genera, several feline gut microorganisms were associated with short-chain fatty acid (SCFAs; e.g., acetate, butyrate, and propionate) synthesis, such as *Faecalibacterium* spp. (Ganz et al., 2022). SCFAs represent the main metabolites produced by gut microbiota fermentation of dietary fibers and non-digestible carbohydrates. They serve as key energy sources for the host epithelium, improve motility, and exert immunomodulatory and anti-inflammatory effects (Yao et al., 2022).

Discrepancies between studies are noticeable and may be partly attributable to various methodologies (Table 1). Most studies rely on sequence-based methods whose results may differ depending on the choice of bacterial targets, such as 16S rRNA and groEL (or *cpn60*) gene sequences. Both genes have been shown to be relevant barcodes for bacteria, with groEL providing a more robust target for species-level identification (Links et al., 2012). Within the numerous studies using 16S rRNA gene sequencing, various amplicon primers, sequencing technologies and bioinformatics tools are also susceptible to impact the assessment of microbial composition (Clooney et al., 2016; O’Sullivan et al., 2021). Of note, group-specific primers were more effective than universal bacterial primers for detection of *Bifidobacterium* and *Lactobacillus* spp. in the feces of healthy pet cats (Ritchie et al., 2010). More advanced techniques such as shotgun metagenomic sequencing are better able to detect less abundant taxa in bacterial communities, and the genera missed by sequence-based methods may carry biologically relevant function (Durazzi et al., 2021). The shotgun metagenomic approach has only been performed in five client-owned healthy cats, thus the representativeness of these results for the overall pet cat population is questionable (Tun et al., 2012). A dysbiosis index has been established to evaluate the balance of intestinal microbiota by measuring the fecal abundances of *Bacteroides*, *Bifidobacterium*, *Clostridium hiranonis*, *Escherichia coli*, *Faecalibacterium*, *Streptococcus*, and *Turicibacter* by quantitative PCR techniques (Sung et al., 2022). It provides a targeted assessment of these bacterial groups that have been determined to be frequently altered in chronic enteropathies or after antibiotic therapies. Thus it depicts the extent of intestinal dysbiosis, and the abundance of *Clostridium hiranonis* also predicts the ability of the intestinal microbiota to convert primary bile acids (Sung et al., 2022). It is now available in routine clinical practice and is also frequently used in the research setting, although it fails to provide in-depth description of the composition of the fecal microbiota. Besides biases introduced by methodological differences, batch effect correction is rarely addressed in the analysis of the gut microbiota from healthy cats. Regarding sample collection and storage, most studies assessed the fecal bacterial community and some surveys were based on the collection and mailing of samples by owners, with risks of inconsistent technical lead times (Ganz et al., 2022). There is a need to reinforce the robustness of the data produced by defining pre-analytical and analytical standards. The feline species lacks a reference gut microbiome gene catalog established by whole genome metagenomics studies, as described in humans and pet dogs (Coelho et al., 2018; Kim et al., 2021). This is a major shortcoming for taxonomic assignment and functional characterization of the healthy feline gut microbiota. It is worth noting that gut microbes are far from being inert and are constantly evolving in response to the different changes in environment and physiology. While such a community plays an essential role in cats’ health, it still can be influenced by many factors including age, dietary habits and exposure to xenobiotics. In this regard, several studies assessed the effect of age on the gut microbiota composition and provided inconsistent results, likely caused by different methodologies, including bacterial culture, 16S rRNA gene sequencing and shotgun sequencing technologies conducted on the feces of colony or client-owned cats (Deusch et al., 2015; Masuoka et al., 2017; Bermingham et al., 2018; Li et al., 2022). For example, the shifts in abundances of *Bifidobacteria* and *Lactobacillus* varied, but both bacterial genera were not found to be predominant in the feline gut microbiota, unlike dogs and humans (Masuoka et al., 2017; Bermingham et al., 2018). Furthermore, the impact of macronutrient composition of pet foods on gut microbiota is a current area of interest. Regarding the effects of dietary complex carbohydrates, two studies conducted on four experimental and 10 privately-owned healthy adult cats respectively, failed to identify significant shifts in microbial communities when adding dietary fiber to the diet (i.e., cellulose, fructo-oligosaccharides (FOS), pectin, and inulin) and showed high inter-individual variability in the response to nutritional modifications (Barry et al., 2012; Garcia-Mazcorro et al., 2017). In a separate study involving eight experimental cats, adding dietary supplements of short-chain FOS, galacto-oligosaccharides (GOS) or a combination of both prebiotics resulted in increased levels of cultured *Bifidobacterium* spp. (Kanakupt et al., 2011), which is known to be associated with gut health (O’Callaghan and van Sinderen, 2016). Cats consuming the diet enriched with mixed short-chain FOS and GOS showed significantly greater fecal concentrations of butyrate and valerate, and a trend toward greater fecal concentrations of acetate (Kanakupt et al., 2011). Of note, Bifidobacteria have been shown to produce acetate, thus being involved in the SCFAs-mediated favorable effects on host gastrointestinal health (Fukuda et al., 2011). This experimental dietary intervention mimics the supplementation with prebiotic fibers that may be implemented in pet cats suffering from gastrointestinal signs. On the other hand, feeding 12 experimental cats with a raw meat-based diet was found to raise the levels of *Clostridium* and *Fusobacterium* in their feces (Butowski et al., 2019). Of note, *Fusobacterium* has been incriminated in the development of cancer in cats and humans (Kostic et al., 2012; Garraway et al., 2018). Addition of plant-based fiber to this high-protein diet induced the predominance of *Prevotella* (similarly to a control industrial pet food) and a group of unclassified *Peptostreptococcaceae* (Butowski et al., 2019). Available data report that *Peptostreptococcaceae* have been involved in the development of various human infections within the digestive tract (Tannock et al., 2012; Wei et al., 2019). However, this bacterial family has been shown to be prominent in eight experimental healthy cats, particularly when fed canned diets, and does not seem to exhibit deleterious effects (Bermingham et al., 2018). Most of the aforementioned dietary interventions have been conducted on small numbers of experimental animals and relied on sequence-based methodologies to assess the gut microbiota. Further studies should be carried out in larger populations of pet cats, and involve the use of whole-genome shotgun sequencing with functional analyses to ensure the consistency and the relevance of these findings. Recent evidence has also shown that lifestyle influences the feline gut mycobiota composition, with indoor cats displaying more *Ascomycota*, while outdoor cats show more *Basidiomycota*. Nevertheless, the presence of the genus *Peniophorella* has been observed in both indoor and outdoor cats and stands out as the dominant component of the mycobiota (Tay et al., 2022). Understanding the influence of different factors on microbial communities would be a prerequisite to better decipher the molecular mechanisms involved in the symbiosis that marks holobiont health (Masuoka et al., 2017; Whittemore et al., 2019; Pilla and Suchodolski, 2021). The dysbiosis index has been shown to remain stable over 2 months in 17 indoor adult pet cats exempt from any living or dietary changes throughout the follow-up period, whereas it showed large shifts in eight client-owned cats receiving antibiotics and in 68 client-owned cats with IBD (Sung et al., 2024). Similar prospective studies including monitoring of the dysbiosis index and deeper assessment of the gut microbiota composition and metabolic activities during nutritional interventions would be desirable to document the impact of physiological factors, that are thought to be minimal when compared with dysbiotic alterations associated with pathological states.

**Table 1:** Methodology of studies assessing the gut microbiota composition in healthy adult cats.

Study	Animals	Samples	Microbiota analysis method
Desai et al. (2009)	9 pet cats	Feces	Cpn60 gene sequencing
Ganz et al. (2022)	161 pet cats	Feces	16S rRNA gene sequencing Universal Primers 505F/816R
Handl et al. (2011)	12 pet cats	Feces	16S rRNA gene pyrosequencing Universal primers 28F/519R
Ritchie et al. (2008)	5 colony cats	Intestinal fragments	16S rRNA gene sequencing Primers 342F/786R
Ritchie et al. (2010)	27 pet cats	Feces	16S rRNA gene sequencing Universal primers 341F/786R + Group-specific primers for *Bifidobacterium* and *Lactobacillus* spp.
Tun et al. (2012)	5 pet cats	Feces	Shotgun 454-pyrosequencing

## 3 Gut microbiota in feline spontaneous IBD and LGITL

Chronic inflammation is thought to arise in genetically susceptible individuals, due to complex interactions between the intestinal immune system, exposome factors (mainly dietary components) and the microbiome (Marsilio, 2021). In populations of pet cats with chronic gastrointestinal signs (i.e., vomiting and/or diarrhea of at least 2–3 weeks’ duration) that did not benefit from exhaustive characterization, bacterial diversity of the fecal microbiota was shown to be decreased when compared with healthy individuals (Suchodolski et al., 2015; Kathrani et al., 2022). The fecal microbiota of 15 partially characterized colony cats with chronic diarrhea, in whom only extraintestinal and infectious/parasitic disorders were ruled out, was predominantly composed of bacteria belonging to the phyla Firmicutes (with prevailing classes *Bacilli* and *Clostridia*), Bacteroidetes (with prevailing classes *Bacteroidia* and *Flavobacteria*), Fusobacteria, Proteobacteria, Tenericutes and Actinobacteria, in decreasing order (Ramadan et al., 2014). Of note, Tenericutes are not major components of the healthy feline gut microbiota and are predominantly commensals or obligate parasites in humans and domestic animals. They are primarily known for their Mollicutes clade, housing opportunistically pathogenic genera like *Mycoplasma*, *Ureaplasma*, and *Acholeplasma* (Trachtenberg, 2005). Alterations in microbial communities from the phylum Firmicutes have been further described in comparison to healthy cats: pet cats with chronic gastrointestinal signs exhibited greater abundance of bacteria from the class *Erysipelotrichia* and the genera *Lactobacillus and Clostridium*, and a decrease in the genus *Faecalibacterium* (Suchodolski et al., 2015; Kathrani et al., 2022). In humans, bacteria from the *Erysipelotrichiaceae* family have been correlated with gastrointestinal inflammation and metabolic disorders (Labbé et al., 2014). *Faecalibacterium* spp. are also commonly decreased in fecal and mucosal samples from human patients with IBD. For instance, *Faecalibacterium prausnitzi*, a strain that has been shown to produce butyrate and exert anti-inflammatory properties, is commonly described as a general health biomarker (Cao et al., 2014). Functionally, qualitative changes in the fecal microbiota of cats with chronic gastrointestinal signs were associated with significant alterations in bacterial gene contents referring to the metabolism of carbohydrates, vitamins, amino acids and xenobiotics (Suchodolski et al., 2015). All these results should be treated with caution, because a more comprehensive characterization of these studied feline populations would be highly desirable, with exhaustive exclusion of extraintestinal illnesses, infectious or parasitic diseases and focal gastrointestinal disorders, with final demonstration of inflammatory or low-grade lymphomatous intestinal infiltration.

**Figure 1:**
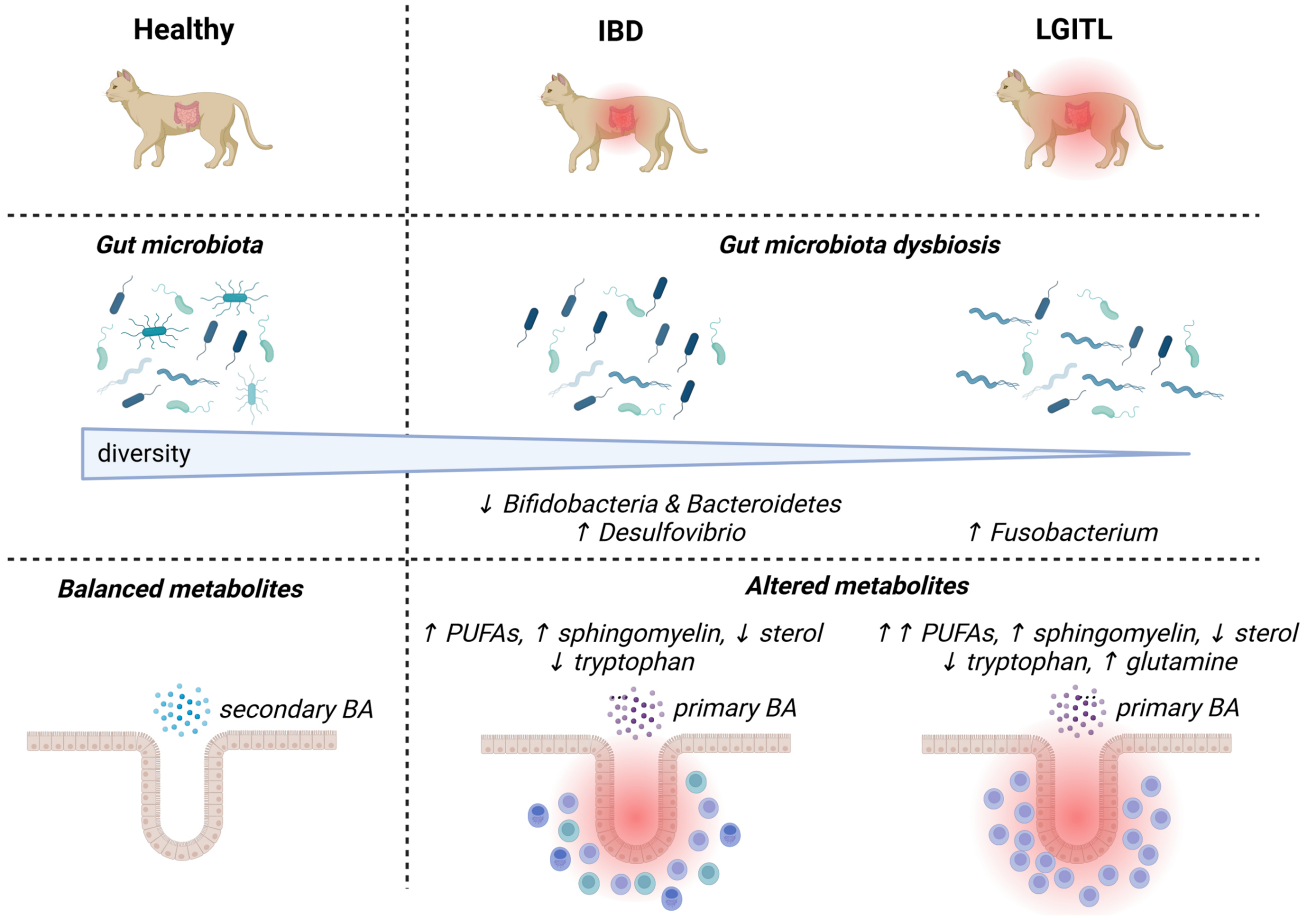
Overview of major changes in the gut microbiota composition and activities in cats with chronic enteropathies (inflammatory bowel diseases and low-grade intestinal T-cell lymphoma). Up and down arrows represent increases and decreases, respectively. BA, bile acids; IBD, inflammatory bowel diseases; LGITL, low-grade intestinal T-cell lymphoma; PUFA, polyunsaturated fatty acid.

Regarding the fecal microbiota of privately-owned cats with histologically confirmed IBD, dysbiotic alterations associated with the disease (Figure 1) are less documented than in humans and dogs. In feline IBD, overall bacterial diversity was shown to be decreased in comparison to healthy cats, but specific patterns of dysbiosis could not be identified in these 13 privately-owned diseased cats (Marsilio et al., 2019). Increased populations of mucosa-associated bacteria, assessed by a more targeted fluorescence *in situ* hybridization (FISH) approach, have been associated with clinical disease activity and duodenal inflammation evaluated by histopathology and cytokine mRNA profiles (Janeczko et al., 2008). Another molecular-based enumeration study using FISH in feline feces showed increased numbers of sulfate-reducing bacteria from the genus *Desulfovibrio* spp., and decreased numbers of bacteria from the genera *Bifidobacteria* spp. and *Bacteroidetes* spp. in colony cats with IBD when compared with healthy colony cats (Inness et al., 2007). However, studies using advanced techniques for in-depth assessment of the gut microbiota in sufficiently large populations of cats with IBD are still lacking. From a functional standpoint, untargeted metabolomic analysis found that metabolic changes identified in cats with IBD were similar to humans and other animal models with IBD. They comprised increased fecal amino-acids consistent with malabsorption, increased fecal arachidonate, omega-3 fatty acids and simple sphingolipids possibly accompanying intestinal inflammation, and decreased fecal indole derivatives attributed to intestinal dysbiosis (Marsilio et al., 2021).

A pathogenic theory that currently prevails in cats is that of a continuum between IBD and LGITL, given the common coexistence of inflammatory and lymphomatous infiltrations, and frequent previous history of IBD in animals with LGITL (Marsilio et al., 2023). Cats with LGITL have been shown to exhibit lower fecal bacterial diversity than healthy cats, and a trend toward lower alpha-diversity than cats with IBD based on 16S rRNA gene sequencing (Figure 1) (Marsilio et al., 2019). This study comparing feces from 14 pet cats with LGITL with feces from 38 privately-owned healthy cats and 13 pet cats with IBD failed to identify significant differences in microbial communities between groups, probably due to low numbers of animals (Marsilio et al., 2019). Targeted bacterial quantification based on FISH in endoscopic or laparoscopic gastrointestinal biopsies identified increased numbers of mucosa-associated *Fusobacterium* spp. and *Bacteroides* spp. in the ileum and increased numbers of mucosa-associated *Fusobacterium* spp. in the colon of 14 pet cats with histologically confirmed LGITL when compared with 14 pet cats with histologic IBD (Garraway et al., 2018). The abundance of *Fusobacteria* spp. in adherent mucus showed the highest correlation with the number of CD11b^+^ myeloid cells and with the up-regulation of NF-κB expression in the gastrointestinal mucosa of cats with LGITL, when compared with cats with IBD (Garraway et al., 2018). A more recent study based on 16S rRNA gene sequencing also identified increased abundance of bacteria from the *Fusobacteriaceae* family in seven pet cats with histologically diagnosed LGITL compared with 13 pet cats with histologic IBD (Benvenuti et al., 2024). Taken together, these findings open the door to a possible implication of *Fusobacterium* spp. in the carcinogenesis of LGITL. Of note, *Fusobacterium* has also been incriminated in carcinogenesis in humans with colorectal neoplasia (Kostic et al., 2012). Functional alterations related to dysbiosis were found to be more severe in 11 pet cats with LGITL than in 11 pet cats with IBD, and fecal concentrations of polyunsaturated fatty acids were shown to discriminate between both types of feline CE (Marsilio et al., 2021). Lower fecal concentrations of indolelactate, a microbial indole catabolite of tryptophan, were also recovered in 31 cats with LGITL when compared with 44 cats with IBD (Barko et al., 2023). These data being scarce, further studies are required to determine whether some characteristics of the fecal microbiota and its metabolic activities may predict the diagnosis of feline IBD or LGITL, and improve therapeutic interventions. In humans, the ITLPD-GI is a recently described entity recognized as a low-grade, clonal, T-cell lymphoproliferative neoplasm arising in the digestive tract (Sanguedolce et al., 2021). It is a rare disease with a protracted clinical course, whose diagnosis is particularly challenging due to heterogeneous histological and molecular features. The relationship between IBD and ITLPD-GI in humans remains unclear and no data are available regarding the implication of gut microbiota in the development of ITLPD-GI. LGITL being a frequent disorder in cats, studying its pathogenesis may provide new insights into the pathogenesis of human ITLPD-GI.

As a whole, the state of knowledge concerning alterations in the intestinal microbiota of cats with CE remains insufficient. Studies are frequently underpowered with small populations of spontaneously ill privately-owned cats (Table 2). Thus, cats with histologically confirmed IBD and LGITL are sometimes grouped together into a broader group of cats with CE to characterize the gut microbiota composition in comparison with healthy cats. A 16S rRNA gene sequencing approach identified a distinct pattern of dysbiosis in 27 pet cats with CE, with increased abundances of facultative anaerobes from the *Enterobacteriaceae* and *Streptococcaceae* families, and decreased abundances of obligate anaerobic members of the phyla Firmicutes (*Ruminococcaceae* and *Turicibacteraceae* families), Actinobacteria (*Bifidobacterium* genus) and Bacteroidetes (*Bacteroides plebius*) (Marsilio et al., 2019). A similar study conducted in 16 pet cats with histologically confirmed CE recovered increased abundances of the phylum Proteobacteria, the orders Enterobacterales and Lactobacillales, the family *Enterobacteriaceae* and the genus *Escherichia Shigella*, and decreased abundances of the phylum Bacteroideta and the order Peptococcales when compared with 14 privately-owned healthy pet cats (Miller et al., 2023). Some of these shifts are similar to those identified by more robust studies conducted in humans with IBD, such as decreased abundances of obligate anaerobes from the Firmicutes and Bacteroidetes phyla (Frank et al., 2007), and increased abundance of facultative anaerobes from the *Enterobacteriaceae* family (Khorsand et al., 2022). Regarding more targeted molecular methods, the dysbiosis index based on the quantification of the abundances of seven select bacterial groups was able to discriminate between groups of 68 cats with CE and 80 healthy pet cats, with 76% of cats with CE presenting increased dysbiosis index (Sung et al., 2022). This biomarker has the advantage of being accessible for the diagnosis of dysbiosis in routine clinical practice. However, it provides partial assessment of the gut microbiota composition and does not bring better insights into the interactions between gut microbiota and feline CE. Metabolic alterations resembling those described in human IBD and relating to tryptophan, arachidonic acid, glutathione and lipids have also been underlined in pet cats with CE (Marsilio et al., 2021; Barko et al., 2023; Miller et al., 2023; Sung et al., 2023). Additional studies are mandatory to relate gut microbiota compositions and metabolic alterations.

**Table 2:** Methodology of studies assessing the gut microbiota composition in cats with suspected or confirmed chronic enteropathies.

Study	Animals	Samples	Microbiota analysis method
Benvenuti et al. (2024)	13 pet cats with IBD; 7 pet cats with LGITL	Feces	16S rRNA gene sequencing Primers for V3-V4 regions (341F/805R)
Garraway et al. (2018)	14 pet cats with IBD; 14 pet cats with LGITL	Intestinal biopsies	Fluorescence *in situ* hybridization Universal bacterial probe Specific probes (*Clostridium* spp., Bacteroides/Prevotella group, *Fusobacterium* spp., Enterobacteriaceae, *Helicobacter* spp., *Faecalibacterium* spp.)
Inness et al. (2007)	11 colony cats with IBD; 34 healthy colony cats	Feces	Fluorescence *in situ* hybridization Specific probes (*Bifidobacterium* spp., *Bacteroides* spp., *C. histolyticum* subgp., *Lactobacillus-Enterococcus* subgp., *Desulfovibrio* spp.)
Janeczko et al. (2008)	17 pet cats with IBD; 10 healthy colony cats	Intestinal biopsies	Fluorescence *in situ* hybridization Universal *bacterial* probe Specific probes (*Clostridium* spp., Bacteroides/Prevotella group, Enterobacteriaceae, *E. coli*, *Streptococcus* spp., *Helicobacter* spp.)
Kathrani et al. (2022)	42 pet cats with suspected or confirmed CE; 14 healthy pet cats	Feces	16S rRNA gene sequencing Primers for V3-V4 regions
Marsilio et al. (2019)	13 pet cats with IBD; 14 pet cats with LGITL; 38 healthy pet cats	Feces	16S rRNA gene sequencing Primers for the V4 region (515F/806R)
Miller et al. (2023)	6 pet cats with IBD; 6 pet cats with LGITL; 6 pet cats with uncharacterized CE; 14 healthy pet cats	Feces	16S rRNA gene sequencing Primers for V3-V4 regions
Ramadan et al. (2014)	15 colony cats with uncharacterized chronic diarrhea	Feces	16S rRNA gene pyrosequencing Primers for the V1-V2 region
Suchodolski et al. (2015)	29 pet cats with uncharacterized chronic diarrhea; 21 healthy pet cats	Feces	16S rRNA gene sequencing Primers for the V4 region (515F/806R)
Sung et al. (2022)	68 pet cats with IBD or LGITL; 80 healthy pet cats	Feces	Quantitative polymerase chain reaction Total bacteria + Specific groups (*Bacteroides*, *Bifidobacterium*, *E. coli*, *Faecalibacterium*, *Fusobacterium*, *Streptococcus*, *Turicibacter*, *Blautia*, *Clostridium hiranonis*)

CE, chronic enteropathy; IBD, histologically confirmed inflammatory bowel disease; LGITL, histologically confirmed low-grade intestinal T-cell lymphoma.

## 4 Modulation of the gut microbiota in cats with CE

Interventional dietary trials (unspecified composition) in 15 experimental cats with chronic diarrhea showed significant correlations between improvement of fecal consistency (assessed by fecal score) and fecal bacterial abundance of the phyla Actinobacteria (i.e., genus *Slackia* and *Collinsella*), Proteobacteria (i.e., *Campylobacter upsaliensis*, the genus *Raoultella*, and unidentified genus of the family Succinivibrionaceae), and Firmicutes (unidentified genus of the family Lachnospiraceae) determined by 16S rRNA 454-pyrosequencing (Ramadan et al., 2014). Feeding a hydrolyzed protein diet to 36 client-owned cats with suspected or histologically confirmed CE induced a decrease in alpha-diversity and an increase in the abundance of *Bifidobacterium* assessed with a 16S rRNA sequencing approach (Kathrani et al., 2022). Various Bifidobacteria strains enhance intestinal barrier functions and regulate cytokine network, reducing therefore intestinal inflammation (Caviglia et al., 2020). Cats that did not respond to a hydrolyzed protein regimen showed higher baseline alpha-diversity and increased abundance of Oscillobacter and Desulfovibrionaceae than responders (Kathrani et al., 2022).

Data regarding the use of probiotics in cats with CE are scarce. In a trial conducted on eight pet cats from the same household suffering from chronic diarrhea, administration of *Bacillus licheniformis*-fermented products improved fecal scores and decreased feline chronic enteropathy activity indexes in some individuals (Lee et al., 2022). Changes in the bacterial composition of the gut microbiota were also described, with a decrease in the abundance of *Clostridium perfringens* and an increase in the abundance of *Blautia* spp., *Ruminococcus torques* and *Ruminococcus gnavus* (Lee et al., 2022). Oral fecal microbiota transplant capsules were prospectively administered to 46 pet cats with chronic gastrointestinal signs, leading to partial stool donor bacterial engraftment (Rojas et al., 2023). The fecal microbiota of responders tended to become more similar to the fecal microbiota of healthy cats (Rojas et al., 2023). All these data determined by 16S rRNA sequencing techniques must be interpreted cautiously because the underlying disorders were not fully investigated and the evolution of gastrointestinal inflammation was not assessed in these populations of cats with suspected CE.

## 5 Conclusion and future prospects

Chronic enteropathies are of increasing interest in companion animals, including in cats, given their challenging diagnosis and multifactorial nature. Recent evidence has questioned the role of the dysbiotic microbiota in such diseases. Still, only a few data are available at this point and it remains a field in its infancy. Characterization of the feline gut microbiota mainly relies on sequence-based approaches and lack more comprehensive evaluation of its composition and activities with deeper shotgun metagenomics studies. Thus, reference data regarding the gut microbiota of healthy cats are lacking. Studies of gut microbiota alterations in cats spontaneously suffering from CE rarely include a sufficient number of histologically confirmed cases of IBD or LGITL. Regarding therapeutic perspectives, microbiota modulation trials are frequently conducted on small populations of experimental animals and fail to sufficiently document beneficial effects on the pathological process of CE. Accordingly, it is now imperative to bear a clear picture of the relevance of the different contributing factors in the development of feline CE and to gain a better mechanistic understanding of microbiota-host interactions in order to open the way to a more individualized medicine.

## Author Contributions

AD: Validation, Writing – original draft, Writing – review & editing. HM: Writing – original draft, Writing – review & editing, Formal Analysis. AK: Writing – original draft, Writing – review & editing, Formal Analysis. VM: Writing – review & editing, Visualization. NA: Writing – review & editing. TM: Writing – review & editing. OS: Writing – review & editing. EM: Writing – review & editing, Conceptualization. JH: Writing – review & editing, Funding acquisition, Writing – original draft. MR: Conceptualization, Writing – review & editing, Funding acquisition, Investigation, Validation, Writing – original draft.
